# Supplementary material for: Xenopus laevis as an infection model for human pathogenic bacteria
Source: Infect Immun. 2025 May 1;93(6):e00126-25. doi: 10.1128/iai.00126-25 (PMC12150759; doi:10.1128/iai.00126-25)
Supplement: Table S1 — Survival data of all infection experiments. [file iai.00126-25-s0001.docx]

**Table S1. Survival rate of frogs**

| strain | bacterial_dose | time | survival | total | exp_date |
| --- | --- | --- | --- | --- | --- |
| NCTC8325-4WT (alive) | 103400000 | 24 | 0 | 5 | 20240416 |
| NCTC8325-4WT (alive) | 34466667 | 24 | 3 | 5 | 20240416 |
| NCTC8325-4WT (alive) | 11488889 | 24 | 5 | 5 | 20240416 |
| NCTC8325-4WT (alive) | 89000000 | 24 | 0 | 5 | 20240417 |
| NCTC8325-4WT (alive) | 29666667 | 24 | 1 | 5 | 20240417 |
| NCTC8325-4WT (alive) | 9888889 | 24 | 5 | 5 | 20240417 |
| NCTC8325-4WT (alive) | 97800000 | 24 | 0 | 5 | 20240418 |
| NCTC8325-4WT (alive) | 32600000 | 24 | 3 | 5 | 20240418 |
| NCTC8325-4WT (alive) | 10866667 | 24 | 5 | 5 | 20240418 |
| NCTC8325-4WT (killed) | 885600000 | 24 | 3 | 5 | 20240416 |
| NCTC8325-4WT (killed) | 295200000 | 24 | 5 | 5 | 20240416 |
| NCTC8325-4WT (killed) | 98400000 | 24 | 5 | 5 | 20240416 |
| NCTC8325-4WT (killed) | 32800000 | 24 | 5 | 5 | 20240416 |
| NCTC8325-4WT (killed) | 10933333 | 24 | 5 | 5 | 20240416 |
| NCTC8325-4WT (killed) | 2192400000 | 24 | 5 | 5 | 20240417 |
| NCTC8325-4WT (killed) | 730800000 | 24 | 5 | 5 | 20240417 |
| NCTC8325-4WT (killed) | 243600000 | 24 | 5 | 5 | 20240417 |
| NCTC8325-4WT (killed) | 7119900000 | 24 | 5 | 5 | 20240418 |
| NCTC8325-4WT (killed) | 2543400000 | 24 | 5 | 5 | 20240418 |
| NCTC8325-4WT (killed) | 842400000 | 24 | 5 | 5 | 20240418 |
| NCTC8325-4WT | 98820000 | 24 | 1 | 5 | 20240313 |
| NCTC8325-4WT | 32940000 | 24 | 4 | 5 | 20240313 |
| NCTC8325-4WT | 10980000 | 24 | 5 | 5 | 20240313 |
| NCTC8325-4WT | 97740000 | 24 | 1 | 5 | 20240318 |
| NCTC8325-4WT | 32580000 | 24 | 2 | 5 | 20240318 |
| NCTC8325-4WT | 10860000 | 24 | 4 | 5 | 20240318 |
| NCTC8325-4WT | 82600000 | 24 | 0 | 5 | 20240414 |
| NCTC8325-4WT | 27533333 | 24 | 1 | 5 | 20240414 |
| NCTC8325-4WT | 9177778 | 24 | 5 | 5 | 20240414 |
| MRSA8 | 1569000000 | 24 | 0 | 5 | 20240423 |
| MRSA8 | 523000000 | 24 | 0 | 5 | 20240423 |
| MRSA8 | 174333333 | 24 | 0 | 5 | 20240423 |
| MRSA8 | 174833333 | 24 | 1 | 5 | 20240424 |
| MRSA8 | 58277778 | 24 | 3 | 5 | 20240424 |
| MRSA8 | 19425926 | 24 | 5 | 5 | 20240424 |
| MRSA8 | 179333333 | 24 | 2 | 5 | 20240425 |
| MRSA8 | 59777778 | 24 | 2 | 5 | 20240425 |
| MRSA8 | 19925926 | 24 | 5 | 5 | 20240425 |
| PAO1 | 743218500 | 24 | 0 | 5 | 20240423 |
| PAO1 | 247739500 | 24 | 0 | 5 | 20240423 |
| PAO1 | 82579833 | 24 | 5 | 5 | 20240423 |
| PAO1 | 688605000 | 24 | 0 | 5 | 20240424 |
| PAO1 | 229535000 | 24 | 0 | 5 | 20240424 |
| PAO1 | 76511667 | 24 | 5 | 5 | 20240424 |
| PAO1 | 807330000 | 24 | 0 | 5 | 20240425 |
| PAO1 | 269110000 | 24 | 0 | 5 | 20240425 |
| PAO1 | 89703333 | 24 | 5 | 5 | 20240425 |
| PAO1 | 826326000 | 24 | 1 | 5 | 20240722 |
| PAO1 | 275442000 | 24 | 3 | 5 | 20240722 |
| PAO1 | 91814000 | 24 | 5 | 5 | 20240722 |
| BAA-2114 | 347724000 | 24 | 5 | 5 | 20240423 |
| BAA-2114 | 115908000 | 24 | 5 | 5 | 20240423 |
| BAA-2114 | 38636000 | 24 | 5 | 5 | 20240423 |
| BAA-2114 | 3370248000 | 24 | 3 | 5 | 20240424 |
| BAA-2114 | 1123416000 | 24 | 5 | 5 | 20240424 |
| BAA-2114 | 374472000 | 24 | 5 | 5 | 20240424 |
| BAA-2114 | 8485803000 | 24 | 0 | 5 | 20240425 |
| BAA-2114 | 2948967000 | 24 | 4 | 5 | 20240425 |
| BAA-2114 | 982989000 | 24 | 3 | 5 | 20240425 |
| EGD | 21200000 | 24 | 1 | 5 | 20240511 |
| EGD | 7066667 | 24 | 2 | 5 | 20240511 |
| EGD | 2355556 | 24 | 5 | 5 | 20240511 |
| EGD | 18400000 | 24 | 2 | 5 | 20240512 |
| EGD | 6133333 | 24 | 4 | 5 | 20240512 |
| EGD | 2044444 | 24 | 4 | 5 | 20240512 |
| EGD | 144000000 | 24 | 1 | 5 | 20240513 |
| EGD | 48000000 | 24 | 1 | 5 | 20240513 |
| EGD | 16000000 | 24 | 5 | 5 | 20240513 |
| NCTC8325-4agr | 434400000 | 24 | 3 | 5 | 20240313 |
| NCTC8325-4agr | 144800000 | 24 | 4 | 5 | 20240313 |
| NCTC8325-4agr | 48266667 | 24 | 5 | 5 | 20240313 |
| NCTC8325-4agr | 1303200000 | 24 | 1 | 5 | 20240318 |
| NCTC8325-4agr | 434400000 | 24 | 5 | 5 | 20240318 |
| NCTC8325-4agr | 144800000 | 24 | 4 | 5 | 20240318 |
| NCTC8325-4agr | 579960000 | 24 | 0 | 5 | 20240325 |
| NCTC8325-4agr | 193320000 | 24 | 3 | 5 | 20240325 |
| NCTC8325-4agr | 64440000 | 24 | 4 | 5 | 20240325 |
| NCTC8325-4agr | 1080000000 | 24 | 0 | 5 | 20240414 |
| NCTC8325-4agr | 360000000 | 24 | 1 | 5 | 20240414 |
| NCTC8325-4agr | 120000000 | 24 | 5 | 5 | 20240414 |
| NCTC8325-4srtA | 96660000 | 24 | 0 | 5 | 20240314 |
| NCTC8325-4srtA | 32220000 | 24 | 3 | 5 | 20240314 |
| NCTC8325-4srtA | 10740000 | 24 | 5 | 5 | 20240314 |
| NCTC8325-4srtA | 75060000 | 24 | 1 | 5 | 20240319 |
| NCTC8325-4srtA | 25020000 | 24 | 4 | 5 | 20240319 |
| NCTC8325-4srtA | 8340000 | 24 | 5 | 5 | 20240319 |
| NCTC8325-4srtA | 73620000 | 24 | 0 | 5 | 20240325 |
| NCTC8325-4srtA | 24540000 | 24 | 3 | 5 | 20240325 |
| NCTC8325-4srtA | 8180000 | 24 | 4 | 5 | 20240325 |
| NCTC8325-4srtA | 95200000 | 24 | 0 | 5 | 20240414 |
| NCTC8325-4srtA | 31733333 | 24 | 3 | 5 | 20240414 |
| NCTC8325-4srtA | 10577778 | 24 | 5 | 5 | 20240414 |
| NCTC8325-4cvfA | 671400000 | 24 | 3 | 5 | 20240314 |
| NCTC8325-4cvfA | 223800000 | 24 | 5 | 5 | 20240314 |
| NCTC8325-4cvfA | 74600000 | 24 | 5 | 5 | 20240314 |
| NCTC8325-4cvfA | 1530900000 | 24 | 1 | 5 | 20240319 |
| NCTC8325-4cvfA | 510300000 | 24 | 2 | 5 | 20240319 |
| NCTC8325-4cvfA | 170100000 | 24 | 5 | 5 | 20240319 |
| NCTC8325-4cvfA | 374220000 | 24 | 2 | 5 | 20240325 |
| NCTC8325-4cvfA | 124740000 | 24 | 5 | 5 | 20240325 |
| NCTC8325-4cvfA | 41580000 | 24 | 4 | 5 | 20240325 |
| NCTC8325-4cvfA | 2000700000 | 24 | 0 | 5 | 20240414 |
| NCTC8325-4cvfA | 732600000 | 24 | 0 | 5 | 20240414 |
| NCTC8325-4cvfA | 244200000 | 24 | 4 | 5 | 20240414 |
| LIPI-1 | 155000000 | 24 | 5 | 5 | 20240511 |
| LIPI-1 | 51666667 | 24 | 5 | 5 | 20240511 |
| LIPI-1 | 17222222 | 24 | 5 | 5 | 20240511 |
| LIPI-1 | 990000000 | 24 | 5 | 5 | 20240512 |
| LIPI-1 | 330000000 | 24 | 4 | 5 | 20240512 |
| LIPI-1 | 110000000 | 24 | 5 | 5 | 20240512 |
| LIPI-1 | 2835000000 | 24 | 5 | 5 | 20240513 |
| LIPI-1 | 900000000 | 24 | 5 | 5 | 20240513 |
| LIPI-1 | 300000000 | 24 | 5 | 5 | 20240513 |
| NCTC8325-4WT | 101400000 | 24 | 4 | 5 | 20241017 |
| NCTC8325-4WT | 33800000 | 24 | 5 | 5 | 20241017 |
| NCTC8325-4WT | 11266667 | 24 | 5 | 5 | 20241017 |
| NCTC8325-4WT | 298800000 | 24 | 0 | 5 | 20241021 |
| NCTC8325-4WT | 99600000 | 24 | 4 | 5 | 20241021 |
| NCTC8325-4WT | 33200000 | 24 | 5 | 5 | 20241021 |
| NCTC8325-4WT | 250800000 | 24 | 0 | 5 | 20241108 |
| NCTC8325-4WT | 83600000 | 24 | 4 | 5 | 20241108 |
| NCTC8325-4WT | 27866667 | 24 | 5 | 5 | 20241108 |
| NCTC8325-4agr | 1294200000 | 24 | 0 | 5 | 20241017 |
| NCTC8325-4agr | 431400000 | 24 | 5 | 5 | 20241017 |
| NCTC8325-4agr | 143800000 | 24 | 5 | 5 | 20241017 |
| NCTC8325-4agr | 1353600000 | 24 | 0 | 5 | 20241021 |
| NCTC8325-4agr | 451200000 | 24 | 5 | 5 | 20241021 |
| NCTC8325-4agr | 150400000 | 24 | 5 | 5 | 20241021 |
| NCTC8325-4agr | 950400000 | 24 | 2 | 5 | 20241108 |
| NCTC8325-4agr | 316800000 | 24 | 5 | 5 | 20241108 |
| NCTC8325-4agr | 105600000 | 24 | 5 | 5 | 20241108 |
| NCTC8325-4hla | 88400000 | 24 | 5 | 5 | 20241017 |
| NCTC8325-4hla | 29466667 | 24 | 5 | 5 | 20241017 |
| NCTC8325-4hla | 9822222 | 24 | 5 | 5 | 20241017 |
| NCTC8325-4hla | 826200000 | 24 | 2 | 5 | 20241021 |
| NCTC8325-4hla | 275400000 | 24 | 4 | 5 | 20241021 |
| NCTC8325-4hla | 91800000 | 24 | 5 | 5 | 20241021 |
| NCTC8325-4hla | 2178900000 | 24 | 0 | 5 | 20241108 |
| NCTC8325-4hla | 709200000 | 24 | 1 | 5 | 20241108 |
| NCTC8325-4hla | 236400000 | 24 | 2 | 5 | 20241108 |
| NCTC8325-4hlb | 92200000 | 24 | 5 | 5 | 20241017 |
| NCTC8325-4hlb | 30733333 | 24 | 5 | 5 | 20241017 |
| NCTC8325-4hlb | 10244444 | 24 | 5 | 5 | 20241017 |
| NCTC8325-4hlb | 873000000 | 24 | 1 | 5 | 20241021 |
| NCTC8325-4hlb | 291000000 | 24 | 4 | 5 | 20241021 |
| NCTC8325-4hlb | 97000000 | 24 | 5 | 5 | 20241021 |
| NCTC8325-4hlb | 694800000 | 24 | 1 | 5 | 20241108 |
| NCTC8325-4hlb | 231600000 | 24 | 3 | 5 | 20241108 |
| NCTC8325-4hlb | 77200000 | 24 | 5 | 5 | 20241108 |
| NCTC8325-4hlahlb | 77600000 | 24 | 5 | 5 | 20241017 |
| NCTC8325-4hlahlb | 25866667 | 24 | 5 | 5 | 20241017 |
| NCTC8325-4hlahlb | 8622222 | 24 | 5 | 5 | 20241017 |
| NCTC8325-4hlahlb | 784800000 | 24 | 5 | 5 | 20241021 |
| NCTC8325-4hlahlb | 261600000 | 24 | 5 | 5 | 20241021 |
| NCTC8325-4hlahlb | 87200000 | 24 | 5 | 5 | 20241021 |
| NCTC8325-4hlahlb | 2373300000 | 24 | 4 | 5 | 20241108 |
| NCTC8325-4hlahlb | 772200000 | 24 | 5 | 5 | 20241108 |
| NCTC8325-4hlahlb | 257400000 | 24 | 5 | 5 | 20241108 |
| BW25113 | 1174500000 | 24 | 5 | 5 | 20250116 |
| BW25113 | 391500000 | 24 | 5 | 5 | 20250116 |
| BW25113 | 130500000 | 24 | 5 | 5 | 20250116 |
| BW25113 | 3304125000 | 24 | 5 | 5 | 20250117 |
| BW25113 | 1001250000 | 24 | 5 | 5 | 20250117 |
| BW25113 | 333750000 | 24 | 5 | 5 | 20250117 |
| BW25113 | 3213000000 | 24 | 5 | 5 | 20250121 |
| BW25113 | 1003500000 | 24 | 5 | 5 | 20250121 |
| BW25113 | 334500000 | 24 | 5 | 5 | 20250121 |
| 168trpC2 | 518850000 | 24 | 5 | 5 | 20250116 |
| 168trpC2 | 172950000 | 24 | 5 | 5 | 20250116 |
| 168trpC2 | 57650000 | 24 | 5 | 5 | 20250116 |
| 168trpC2 | 1627425000 | 24 | 4 | 5 | 20250117 |
| 168trpC2 | 494550000 | 24 | 5 | 5 | 20250117 |
| 168trpC2 | 164850000 | 24 | 5 | 5 | 20250117 |
| 168trpC2 | 993600000 | 24 | 4 | 5 | 20250121 |
| 168trpC2 | 346050000 | 24 | 5 | 5 | 20250121 |
| 168trpC2 | 115350000 | 24 | 5 | 5 | 20250121 |

| exp_date | bacterial_dose |  | Time after injection and the number of surviving frogs | | | | | | |
| --- | --- | --- | --- | --- | --- | --- | --- | --- | --- |
|  |  |  | 0 | 12 | 24 | 36 | 48 | 60 | 72 |
| 20240620 | 97800000 | NCTC8325-4×1 | 5 | 1 | 0 | 0 | 0 | 0 | 0 |
|  | 32600000 | NCTC8325-4×0.33 | 5 | 4 | 1 | 1 | 1 | 1 | 1 |
|  | 10866667 | NCTC8325-4×0.11 | 5 | 4 | 4 | 4 | 4 | 4 | 4 |
|  | 0 | PBS | 5 | 5 | 5 | 5 | 5 | 5 | 5 |
| 20240625 | 94600000 | NCTC8325-4×1 | 5 | 5 | 4 | 4 | 4 | 4 | 4 |
|  | 31533333 | NCTC8325-4×0.33 | 5 | 4 | 4 | 4 | 4 | 4 | 4 |
|  | 10511111 | NCTC8325-4×0.11 | 5 | 5 | 5 | 5 | 5 | 5 | 5 |
|  | 0 | PBS | 5 | 5 | 5 | 5 | 5 | 5 | 5 |
| 20240626 | 88200000 | NCTC8325-4×1 | 5 | 2 | 0 | 0 | 0 | 0 | 0 |
|  | 29400000 | NCTC8325-4×0.33 | 5 | 5 | 4 | 3 | 3 | 3 | 3 |
|  | 9800000 | NCTC8325-4×0.11 | 5 | 5 | 5 | 5 | 5 | 5 | 5 |
|  | 0 | PBS | 5 | 5 | 5 | 5 | 5 | 5 | 5 |
| 20240724 | 95600000 | NCTC8325-4×1 | 5 | 5 | 2 | 2 | 2 | 2 | 2 |
|  | 31866667 | NCTC8325-4×0.33 | 5 | 5 | 4 | 4 | 4 | 4 | 4 |
|  | 10622222 | NCTC8325-4×0.11 | 5 | 5 | 5 | 5 | 5 | 5 | 5 |
|  | 0 | PBS | 5 | 5 | 5 | 5 | 5 | 5 | 5 |

| exp_date | bacterial_dose |  | Time after injection and the number of surviving frogs | | | | | | | | | | |
| --- | --- | --- | --- | --- | --- | --- | --- | --- | --- | --- | --- | --- | --- |
|  |  |  | 0 | 12 | 24 | 36 | 48 | 60 | 72 | 84 | 96 | 108 | 120 |
| 20240509 | 101600000 | NCTC8325-4×1 + KM | 5 | 5 | 5 | 5 | 5 | 5 | 5 | 5 | 5 | 5 | 5 |
|  |  | NCTC8325-4×1 + OX | 5 | 5 | 5 | 3 | 2 | 2 | 2 | 2 | 2 | 2 | 2 |
|  |  | NCTC8325-4×1 + VCM | 5 | 5 | 5 | 5 | 5 | 5 | 5 | 5 | 5 | 5 | 5 |
|  |  | NCTC8325-4×1 + PBS | 5 | 2 | 1 | 1 | 1 | 1 | 1 | 1 | 1 | 1 | 1 |
|  | 481000000 | MRSA8×1 + KM | 5 | 2 | 0 | 0 | 0 | 0 | 0 | 0 | 0 | 0 | 0 |
|  |  | MRSA8×1 + OX | 5 | 5 | 0 | 0 | 0 | 0 | 0 | 0 | 0 | 0 | 0 |
|  |  | MRSA8×1 + VCM | 5 | 5 | 5 | 5 | 5 | 5 | 5 | 5 | 5 | 5 | 5 |
|  |  | MRSA8×1 + PBS | 5 | 2 | 0 | 0 | 0 | 0 | 0 | 0 | 0 | 0 | 0 |
|  |  | PBS + PBS | 5 | 5 | 5 | 5 | 5 | 5 | 5 | 5 | 5 | 5 | 5 |
| 20240521 | 108000000 | NCTC8325-4×1 + KM | 5 | 5 | 5 | 5 | 5 | 5 | 5 | 5 | 5 | 5 | 5 |
|  |  | NCTC8325-4×1 + OX | 5 | 5 | 4 | 3 | 2 | 2 | 2 | 2 | 2 | 2 | 2 |
|  |  | NCTC8325-4×1 + VCM | 5 | 5 | 5 | 4 | 4 | 4 | 4 | 4 | 4 | 4 | 4 |
|  |  | NCTC8325-4×1 + PBS | 5 | 2 | 0 | 0 | 0 | 0 | 0 | 0 | 0 | 0 | 0 |
|  | 743500000 | MRSA8×1 + KM | 5 | 1 | 1 | 1 | 1 | 1 | 1 | 1 | 1 | 1 | 1 |
|  |  | MRSA8×1 + OX | 5 | 0 | 0 | 0 | 0 | 0 | 0 | 0 | 0 | 0 | 0 |
|  |  | MRSA8×1 + VCM | 5 | 5 | 5 | 5 | 5 | 5 | 5 | 5 | 5 | 5 | 5 |
|  |  | MRSA8×1 + PBS | 5 | 0 | 0 | 0 | 0 | 0 | 0 | 0 | 0 | 0 | 0 |
|  |  | PBS + PBS | 5 | 5 | 5 | 5 | 5 | 5 | 5 | 5 | 5 | 5 | 5 |
| 20240527 | 96000000 | NCTC8325-4×1 + KM | 5 | 5 | 5 | 5 | 5 | 5 | 5 | 5 | 5 | 5 | 5 |
|  |  | NCTC8325-4×1 + OX | 5 | 5 | 4 | 4 | 4 | 4 | 4 | 4 | 4 | 4 | 4 |
|  |  | NCTC8325-4×1 + VCM | 5 | 5 | 5 | 5 | 5 | 5 | 5 | 5 | 5 | 5 | 5 |
|  |  | NCTC8325-4×1 + PBS | 5 | 2 | 1 | 1 | 1 | 1 | 1 | 0 | 0 | 0 | 0 |
|  | 491500000 | MRSA8×1 + KM | 5 | 1 | 0 | 0 | 0 | 0 | 0 | 0 | 0 | 0 | 0 |
|  |  | MRSA8×1 + OX | 5 | 2 | 0 | 0 | 0 | 0 | 0 | 0 | 0 | 0 | 0 |
|  |  | MRSA8×1 + VCM | 5 | 5 | 5 | 5 | 5 | 5 | 5 | 5 | 5 | 5 | 5 |
|  |  | MRSA8×1 + PBS | 5 | 0 | 0 | 0 | 0 | 0 | 0 | 0 | 0 | 0 | 0 |
|  |  | PBS + PBS | 5 | 5 | 5 | 5 | 5 | 5 | 5 | 5 | 5 | 5 | 5 |

| exp_date | bacterial_dose |  | Time after injection and the number of surviving frogs | | | | | | | | | | |
| --- | --- | --- | --- | --- | --- | --- | --- | --- | --- | --- | --- | --- | --- |
|  |  |  | 0 | 12 | 24 | 36 | 48 | 60 | 72 | 84 | 96 | 108 | 120 |
| 20240516 | 276233500 | PAO1×0.33 + KM in PBS | 5 | 5 | 5 | 5 | 5 | 5 | 5 | 5 | 5 | 5 | 5 |
|  |  | PAO1×0.33 + CPFX in MilliQ | 5 | 5 | 5 | 5 | 5 | 5 | 5 | 4 | 4 | 4 | 4 |
|  |  | PAO1×0.33 + CAZ in PBS | 5 | 5 | 5 | 5 | 5 | 4 | 4 | 4 | 4 | 4 | 4 |
|  |  | PAO1×0.33 + PBS | 5 | 4 | 0 | 0 | 0 | 0 | 0 | 0 | 0 | 0 | 0 |
|  |  | PAO1×0.33 + MilliQ | 5 | 4 | 2 | 1 | 1 | 1 | 1 | 1 | 1 | 1 | 1 |
|  | 6697508143 | BAA2114×27 + KM in PBS | 5 | 4 | 2 | 0 | 0 | 0 | 0 | 0 | 0 | 0 | 0 |
|  |  | BAA2114×27 + CPFX in MilliQ | 5 | 5 | 4 | 3 | 2 | 2 | 2 | 2 | 2 | 2 | 2 |
|  |  | BAA2114×27 + CAZ in PBS | 5 | 4 | 3 | 2 | 1 | 1 | 1 | 1 | 1 | 1 | 1 |
|  |  | BAA2114×27 + PBS | 5 | 4 | 0 | 0 | 0 | 0 | 0 | 0 | 0 | 0 | 0 |
| 20240529 | 391792500 | PAO1×0.33 + KM in PBS | 5 | 5 | 4 | 4 | 4 | 4 | 4 | 4 | 4 | 4 | 4 |
|  |  | PAO1×0.33 + CPFX in MilliQ | 5 | 5 | 5 | 5 | 5 | 5 | 5 | 5 | 5 | 5 | 5 |
|  |  | PAO1×0.33 + CAZ in PBS | 5 | 5 | 5 | 4 | 2 | 2 | 2 | 2 | 2 | 2 | 2 |
|  |  | PAO1×0.33 + PBS | 5 | 4 | 0 | 0 | 0 | 0 | 0 | 0 | 0 | 0 | 0 |
|  | 16520233500 | BAA2114×27 + KM in PBS | 5 | 2 | 2 | 0 | 0 | 0 | 0 | 0 | 0 | 0 | 0 |
|  |  | BAA2114×27 + CPFX in MilliQ | 5 | 2 | 2 | 1 | 1 | 1 | 1 | 1 | 1 | 1 | 1 |
|  |  | BAA2114×27 + CAZ in PBS | 5 | 0 | 0 | 0 | 0 | 0 | 0 | 0 | 0 | 0 | 0 |
|  |  | BAA2114×27 + PBS | 5 | 3 | 2 | 1 | 0 | 0 | 0 | 0 | 0 | 0 | 0 |
|  |  | PBS + PBS | 5 | 5 | 5 | 5 | 5 | 4 | 4 | 4 | 4 | 4 | 4 |
| 20240610 | 229535000 | PAO1×0.33 + KM in PBS | 5 | 5 | 5 | 4 | 4 | 4 | 4 | 4 | 4 | 4 | 4 |
|  |  | PAO1×0.33 + CPFX in MilliQ | 5 | 5 | 5 | 5 | 5 | 5 | 5 | 5 | 5 | 5 | 5 |
|  |  | PAO1×0.33 + CAZ in PBS | 5 | 5 | 5 | 5 | 5 | 4 | 4 | 4 | 4 | 4 | 3 |
|  |  | PAO1×0.33 + PBS | 5 | 3 | 0 | 0 | 0 | 0 | 0 | 0 | 0 | 0 | 0 |
|  | 13225214250 | BAA2114×27 + KM in PBS | 5 | 2 | 0 | 0 | 0 | 0 | 0 | 0 | 0 | 0 | 0 |
|  |  | BAA2114×27 + CPFX in MilliQ | 5 | 5 | 3 | 2 | 1 | 1 | 1 | 1 | 1 | 1 | 1 |
|  |  | BAA2114×27 + CAZ in PBS | 5 | 3 | 0 | 0 | 0 | 0 | 0 | 0 | 0 | 0 | 0 |
|  |  | BAA2114×27 + PBS | 5 | 1 | 0 | 0 | 0 | 0 | 0 | 0 | 0 | 0 | 0 |
|  |  | PBS + PBS | 5 | 5 | 5 | 5 | 5 | 5 | 5 | 5 | 5 | 5 | 5 |

| exp_date | bacterial_dose |  | Time after injection and the number of surviving frogs | | | | | | | | | | |
| --- | --- | --- | --- | --- | --- | --- | --- | --- | --- | --- | --- | --- | --- |
|  |  |  | 0 | 12 | 24 | 36 | 48 | 60 | 72 | 84 | 96 | 108 | 120 |
| 20240612 | 0 | KM in PBS | 5 | 5 | 5 | 5 | 5 | 5 | 5 | 5 | 5 | 5 | 5 |
|  |  | OX in PBS | 5 | 5 | 5 | 5 | 5 | 5 | 5 | 5 | 5 | 5 | 5 |
|  |  | VCM in PBS | 5 | 5 | 5 | 5 | 5 | 5 | 5 | 5 | 5 | 5 | 5 |
|  |  | CPFX in MilliQ | 5 | 5 | 5 | 5 | 5 | 5 | 5 | 5 | 5 | 5 | 5 |
|  |  | CAZ in PBS | 5 | 5 | 5 | 5 | 5 | 5 | 5 | 5 | 5 | 5 | 5 |
|  |  | PBS | 5 | 5 | 5 | 5 | 5 | 5 | 5 | 5 | 5 | 5 | 5 |
